# A new nanophyine genus, *Zhangius* gen. nov., with descriptions of two new species (Coleoptera, Brentidae, Nanophyinae) from China and Thailand

**DOI:** 10.3897/zookeys.1273.175356

**Published:** 2026-03-18

**Authors:** Zhiliang Wang, Miguel A. Alonso-Zarazaga

**Affiliations:** 1 Museum of Beijing Forestry University, Beijing, China Museo Nacional de Ciencias Naturales (CSIC) Madrid Spain; 2 Colección de Entomología, Museo Nacional de Ciencias Naturales (CSIC), José Gutiérrez Abascal, 2, E-28006 Madrid, Spain Museum of Beijing Forestry University Beijing China

**Keywords:** Chiang Mai, identification key, *
Lyalia
*, new genus, *
Shiva
*, taxonomy, weevils, Yunnan

## Abstract

A new nanophyine genus, *Zhangius* Wang & Alonso-Zarazaga, **gen. nov**., with two new species *Zhangius rhymbon* Wang & Alonso-Zarazaga, **sp. nov**. (type species) and *Zhangius lophos* Wang & Alonso-Zarazaga, **sp. nov**., are described. The diagnostic characters of the new genus and species are provided. Keys to the male and female of the species and host data are presented.

## Introduction

The latest taxon counts of the family Nanophyidae Gistel, 1848 (Alonso-Zarazaga and Lyal 1999; Alonso-Zarazaga and Perrin 2011), or subfamily Nanophyinae Gistel, 1848 (Oberprieler et al. 2007; McKenna et al. 2009; Alonso-Zarazaga 2014; Alonso-Zarazaga et al. 2023) of the family Brentidae Billberg, 1820 are 33 genera and approximately 310 described species (Alonso-Zarazaga 2014). Seven new genera have been described sporadically in the 25 years since Alonso-Zarazaga (1989) erected the taxonomic system and provided a preliminary phylogenetic framework, based on western Palearctic fauna (*Austronanodes* Zimmerman, 1993, *Meregallia* Alonso-Zarazaga, 1990a, *Damnux* Lyal & Curran, 2003, *Indophyes* Friedman, 2012, *Chibizo* Kantoh & Kojima, 2011, *Lyalia* Alonso-Zarazaga & Perrin, 2011, *Kantohia* Alonso-Zarazaga & Perrin, 2011). Other than *Austronanodes*, all genera were originally described from the Oriental region, which remains a relative taxonomic gap for this group. Furthermore, in Nanophyinae, most of the known host species are associated with herbs or shrubs, but only a few species (e.g., *Damnux*) live in the canopies of dipterocarp trees in the rainforest (Lyal and Curran 2003).

**Figures 1–5.**
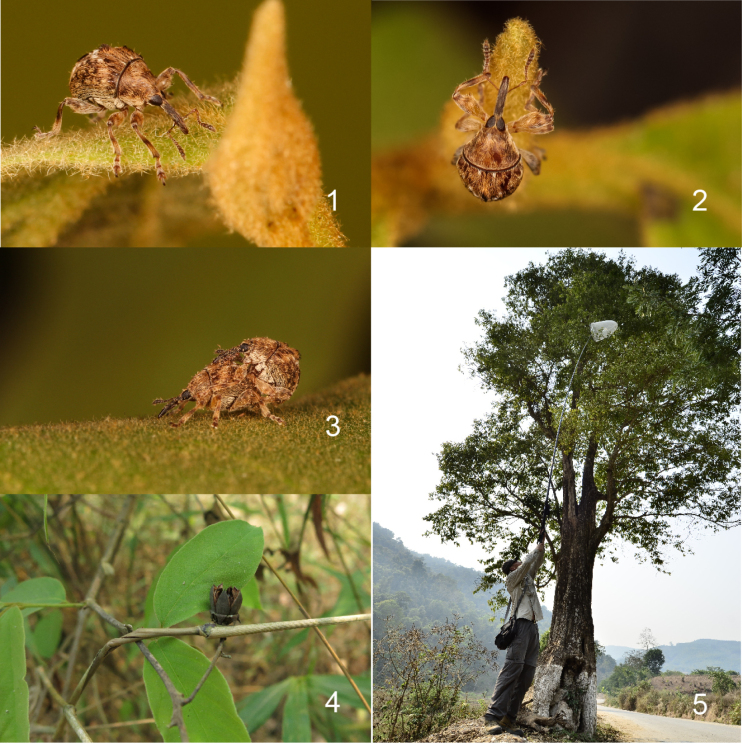
*Zhangius rhymbon* Wang & Alonso-Zarazaga, sp. nov. **1**. Male; **2**. Female; **3**. Mating; **4**. Host plant, *Lagerstroemia tomentosa* C. Presl, 1844, leaves and capsule; **5**. Crown collecting by long-handle tropical net.

In 2010, we were allowed to select weevils from a special collection of Prof. Li Shuqiang (IOZ, CAS), who is working on spiders. The specimens were collected by using a spray machine to apply pesticide to tree canopies in Xishuangbanna, Yunnan. We found several nanophyid specimens which could be morphologically sorted into two allied species, but they did not belong to any hitherto known genus. Also, an unpublished molecular analysis of nanophyine weevils, encompassing most genera in this group, indicates that these two species diverged from a common node, forming a clade distinct from other genera (Xuankun Li et al., unpublished). When we returned to the locality we found that one of the two species lives on *Lagerstroemia tomentosa* C. Presl, 1844 (Lythraceae) (Figs 1–4), which is 20–30 m tall (Qin and Graham 2007) with the lowest branch at least 5 m above the ground. Additionally, a series of Thailand specimens borrowed from Prof. Marek Wanat in 2014 represents one of the two species mentioned. Thus, to place these two new species, we describe a new genus in the present paper.

## Material and methods

### Collecting method

Specimens were collected from the canopy of the plant *L. tomentosa* by using a canopy pesticide spray and a “Lito” tropical net with a 9 m length handle (Fig. 5).

### Type depositories

**IZCAS** (Institute of Zoology, Chinese Academy of Sciences, Beijing, China), **MNCN** (Museo Nacional de Ciencias Naturales, Madrid, Spain), **MNHW** (private collection, M. Wanat, Wrocław, Poland), **MBFU** (Museum of Beijing Forestry University, Beijing, China), **MNHN** (Muséum national d’Histoire naturelle, Paris, France), **SMTD** (Senckenberg Museum für Tierkunde Dresden, Germany).

### Taxonomy

The dissecting method used follows Alonso-Zarazaga (1990a). The abdomen was soaked overnight in lukewarm 10% sodium hydroxide to digest soft tissues. Genitalia and terminalia were photographed in glycerine and later mounted in DMHF (5, 5-dimethyl-hydantoin formaldehyde resin) on an acetate card, pinned together with the tergites and sternites.

Descriptions were made using a binocular Nikon SMZ 1500 microscope. Photographs (Figs 1, 2, 3, 4, 5, 6, 7, 8, 9, 36) were taken with a Canon EOS 7D Mark II, 10–11 with a FEI ESEM Quanta 450 device controlled by the xT microscope software. Extended focus images (Figs 6, 7, 8, 9) were generated with Combine ZP 7.0 by Alan Hadley and edited with Adobe Photoshop CS 6.0 if required. Fig. 37 was obtained using a Xradia micro-CT 400. Drawings (Figs 12, 13, 14, 15, 16, 17, 18, 19, 20, 21, 22, 23, 24, 25, 26, 27, 28, 29, 30, 31, 32, 33, 34, 35) were made from the original photographs using Adobe Illustrator CS6.0 or directly with a drawing tube attached to a Leica DM 2500 microscope.

**Figures 6, 7.**
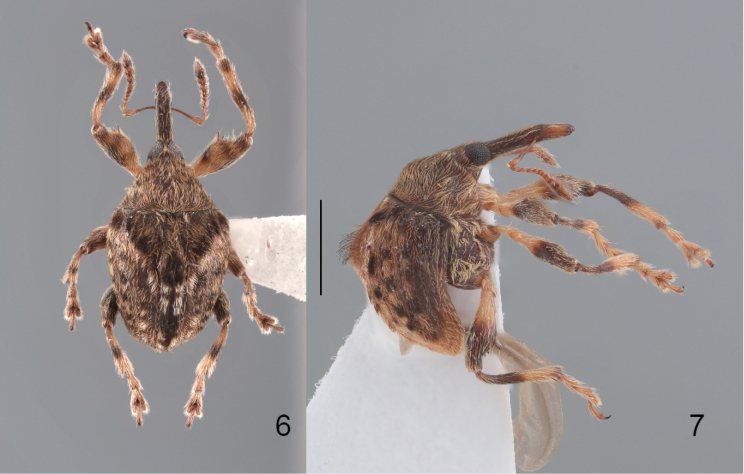
*Zhangius rhymbon* Wang & Alonso-Zarazaga, sp. nov. **6**. Dorsal view; **7**. Lateral view.

**Figures 8, 9.**
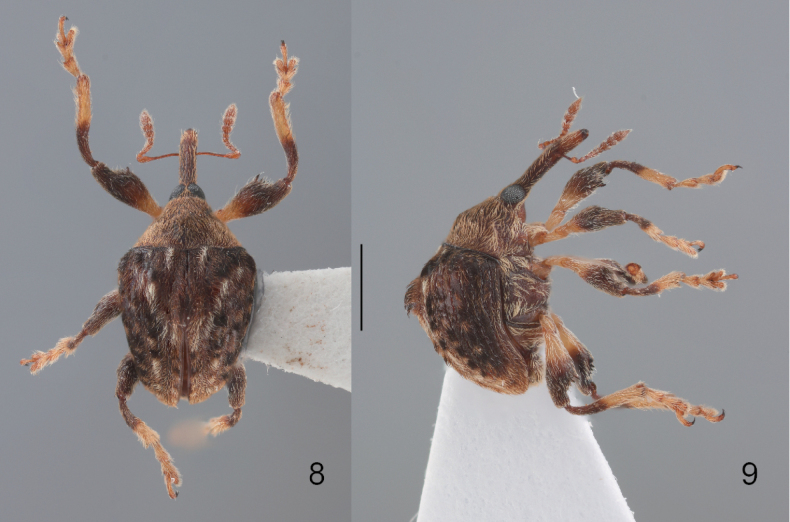
*Zhangius lophos* Wang & Alonso-Zarazaga, sp. nov. **8**. Dorsal view; **9**. Lateral view.

**Figures 10, 11.**
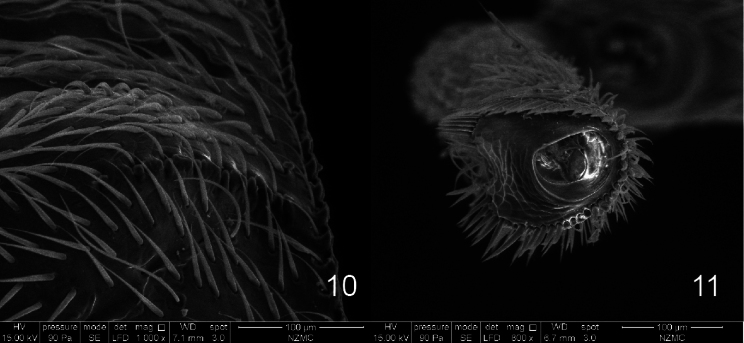
*Zhangius rhymbon*. **10**. Interstria 8 basally crenulate-keeled; **11**. Hind tibia without mucro.

**Figures 12–15.**
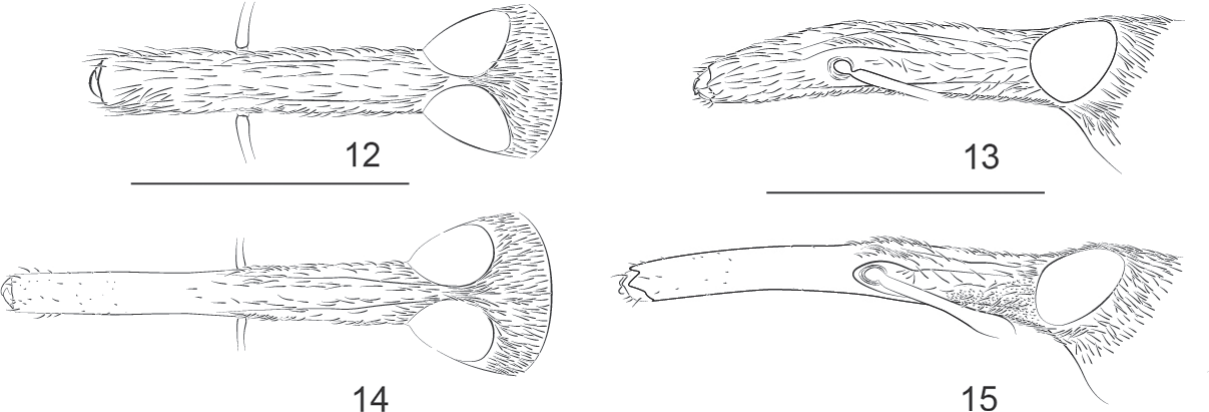
Rostrum of *Z. lophos*. **12**. Male, dorsal view; **13**. Male, lateral view; **14**. Female, dorsal view; **15**. Female, lateral view.

**Figures 16–19.**
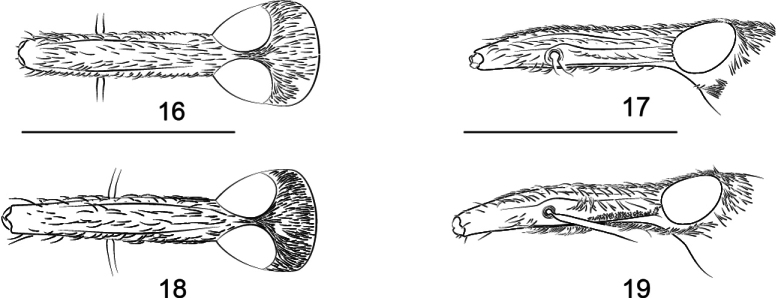
Rostrum of *Z. rhymbon*. **16**. Male, dorsal view; **17**. Male, lateral view; **18**. Female, dorsal view; **19**. Female lateral view.

**Figures 20–27.**
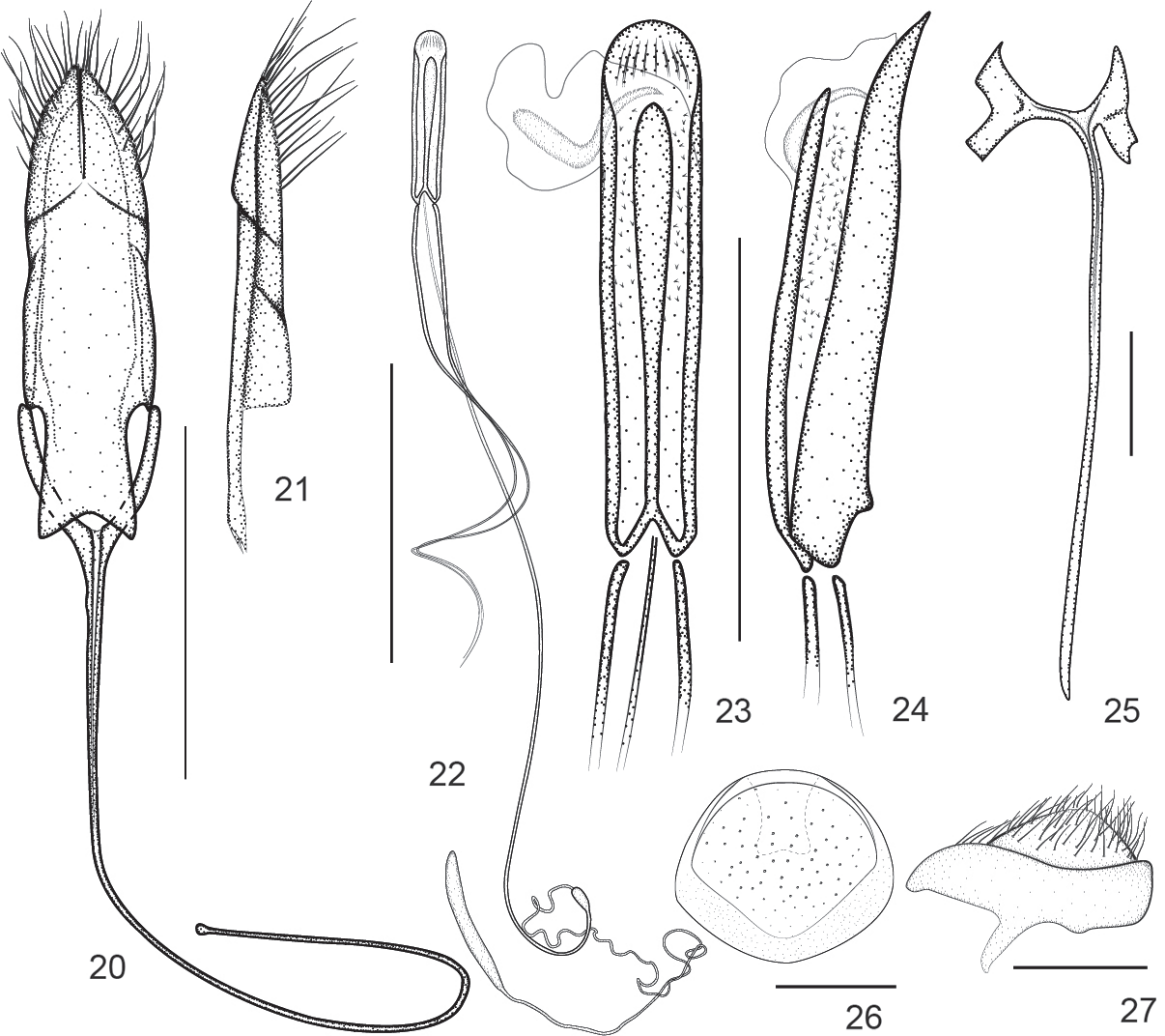
Genitalia of *Zhangius rhymbon*. **20**. Tegmen, dorsal view; **21**. Tegminal plate, lateral view; **22**. Aedeagus, general view; **23**. Aedeagal tube, dorsal view; **24**. Aedeagal tube, lateral view; **25**. Spiculum gastrale, dorsal view; **26**. Pygidium, dorsal view; **27**. Pygidium, lateral view.

**Figures 28–34.**
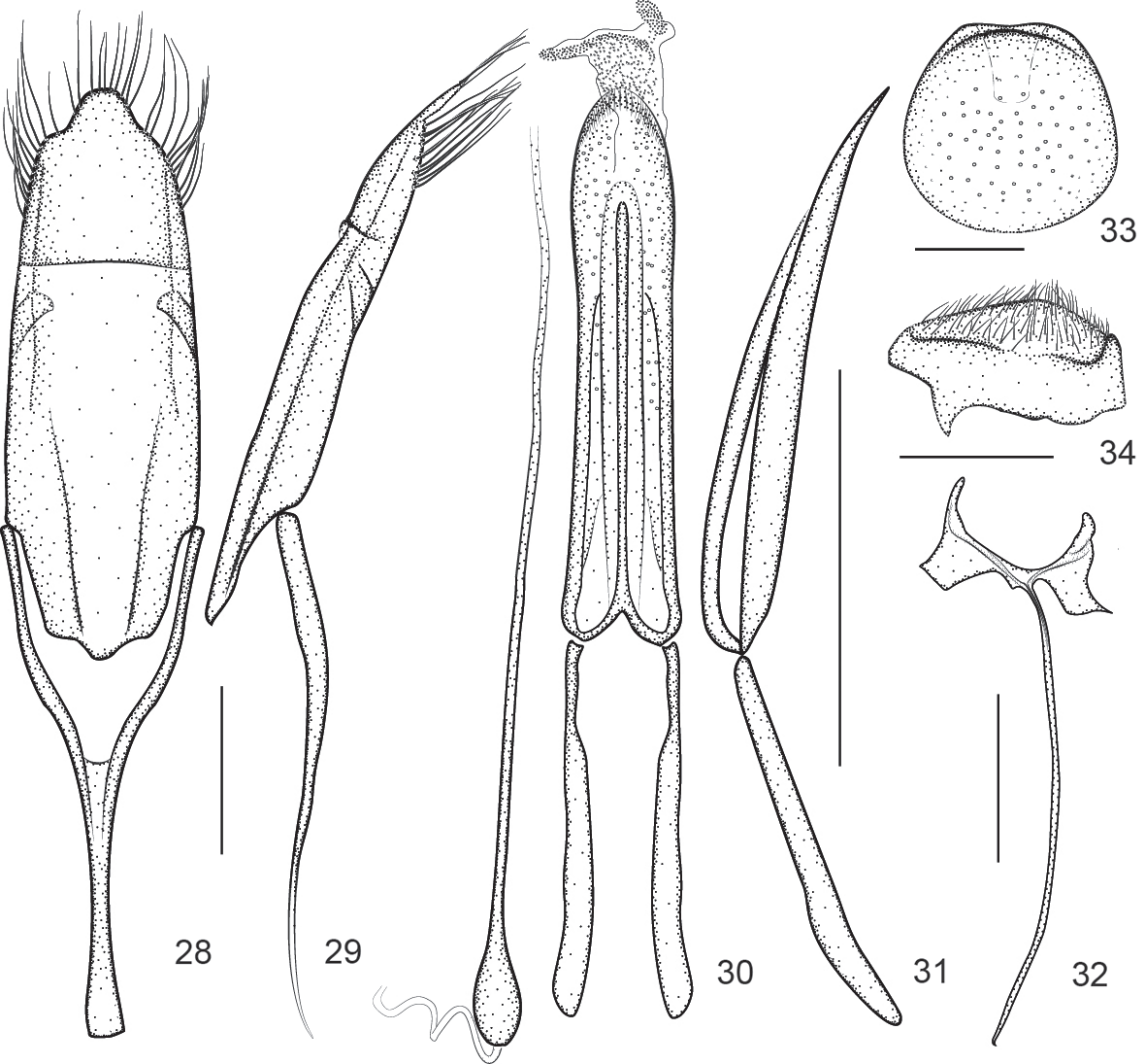
Genitalia of *Zhangius lophos*. **28**. Tegmen, dorsal view; **29**. Tegmen, lateral view; **30**. Aedeagus, dorsal view (flagellum shown separately on the left); **31**. Aedeagus, lateral view; **32**. Spiculum gastrale, dorsal view; **33**. Pygidium, dorsal view; **34**. Pygidium, lateral view.

**Figures 35–37.**
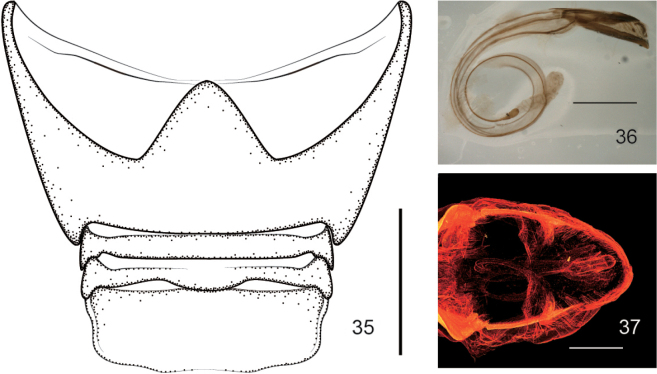
*Zhangius rhymbon*. **35**. Abdominal plate; **36, 37**. Form of the penis in the abdomen.

Labels have been written as originally, in Chinese script. Added transliterations into Pinyin, or translations are placed in square brackets. Labels are separated by two slashes (//) and lines within a label by one slash (/). All the labels in Chinese carry in their lower margin 中国科学院动物研究所 [Zhōngguó kēxuéyuàn dòngwù yánjiū suǒ = Chinese Academy of Sciences, Institute of Zoology].

Nomenclature of the rostral parts follows Alonso-Zarazaga (1989), and that of genitalia follows Alonso-Zarazaga (1990b).

## Taxonomic treatment

### Zhangius

Taxon classificationAnimaliaColeopteraBrentidae

Wang & Alonso-Zarazaga gen. nov.

0FB44A64-B1D9-5AB0-B92E-3923B9046B3F

https://zoobank.org/810B97CD-9686-4F06-ABCC-8674998CEAE6

#### Type species.

*Zhangius rhymbon* Wang & Alonso-Zarazaga, sp. nov. Gender masculine.

#### Diagnosis.

The diagnostic characters that can be used to distinguish *Zhangius* from other genera with 6 desmomeres in the antennae are as follows:

***Vestiture***. Elytra with dense lanceolate setae, some black elongate setae arranged in fascicles located at interstriae; each elytron with 5 suberect blackish fascicled scales respectively on interstriae 1, 3, 4, 5 and 7 (or 1, 3 and 7) at basal area, generally arranged in a V-shaped band.

***Rostrum***. In lateral view, rostral base not forming an angle with frons, male prorostrum rather short, tapering apicad, female prorostrum relatively distinct, elongate and glabrous.

***Antenna***. Funicle with 6 desmomeres, desmomere 2 as long as desmomere 1.

***Head***. Eyes not touching on forehead.

***Thorax***. In lateral view, pronotum distinctly angled at middle; in dorsal view, extremely widened at base.

***Elytra***. Dorsal surface with tubercles at base of medial interstriae 1, 3, 4, 5; those on middle interstria larger than others.

***Legs***. Male metatibiae not mucronate.

***Genitalia***. Flagellum rather to extremely elongate, at least 2× longer than aedeagal tube; apical margin of male pygidium with a tongue-like process (Figs 26, 33), being an abrupt internal projection of apical pygidium margin; fenestrae variable.

#### Description.

***Integument*** with dorsal part generally pale to dark brown, ventral part more or less paler than dorsum, legs yellowish with brown to dark brown marks, meso- and metarostrum dark brown with shiny blackish keels, prorostrum yellowish to reddish, antennae yellowish brown.

***Vestiture*** composed of whitish, yellowish to brownish and black, lanceolate scales, those on antennae and legs narrower than elsewhere; rostrum with dense scales, especially elongate and narrower laterally on prorostrum; suberect blackish scales arranged in fascicles on interstriae 1, 3, 4, 5 and 7 respectively (but in *Z. lophos* interstriae 4 and 5 basally normal, 3 with more black fascicled scales at middle), fascicle on interstriae 1 distinctly elongate, located at about basal 1/3 of elytra, apex of scales curved backward; middle area of venter including inner sides of coxae with soft elongate hairs, especially dense on ventrites; specialized setae present on odd interstriae and pronotum, short.

***Rostrum*** subcylindrical, or subrectangular in section; in dorsal view: widest at mesorostrum; prorostrum slightly constricted apicad, apex shiny with tiny punctures; mesorostrum with two strong dorsal submedial keels, protuberant and shiny, dorsal submedial sulci wide, sparsely punctate; metarostrum with sides almost parallel, dorsal submedial sulci and dorsal submedial keels weak, longitudinally wrinkled with irregular short keels; in lateral view: nearly straight, prorostrum sides converging to apex in male, while more tapering and elongate in female; keels and sulci clear, scrobe enlarged and almost fused with false scrobe, parallel with rostrum.

***Antennae*** inserted at 1/4–1/3 of rostrum in male, before or behind middle in female; funicle with 6 desmomeres, desmomere 1 symmetrical, desmomeres 2–6 slightly asymmetrical; scape a little longer than desmomeres and club respectively, not reaching level of front margin of eye in resting position; desmomere 2 as long as 3, slightly shorter than desmomere 1, slightly longer than desmomere 4, 5 and 6, desmomeres 1–3 elongate, desmomeres 4–5 transverse; club loose, segments symmetrical, about as long as desmomeres.

***Head*** subconical, almost as long as wide; frons flat, not forming an angle with metarostrum in lateral view; eyes subrounded, flat, not touching in dorsal midline.

***Pronotum*** trapezoid, moderately transverse, base approximately twice as wide as apex, apical margin straight, basal margin slightly projecting posteriad at middle; in dorsal view slightly widened at middle, in lateral view, clearly prominent at middle, nearly forming an obtuse angle; discal punctures dense and fine.

***Elytra*** elongate, triangular, evidently constricted apicad, in dorsal view maximum width across humeri; dorsal surface with tubercles at base of interstriae 1, 3, 4, 5, humeri at interstriae 7 also developed; tubercle at interstria 1 elongate along elytral suture, larger than others, located at basal 1/3 of elytra; striae shallow but clear, punctate, stria 10 complete; interstriae flat with fine punctures, interstria 8 basally with crenulate keel, not surpassing humeri (Fig. 10).

***Ventral areas***. Procoxae projecting, subconical, tangential with respect to each other. Mesocoxae separated by a distance less than 1/2 the transverse diameter of mesocoxa. Abdominal ventrites (Fig. 36) densely and finely punctate, suture I invisible, suture IV complete, ventrites 3–5 midlaterally foveate, foveae of ventrite 3 reach to sides, foveae of ventrite 4 widened and conjoint medially, foveae of ventrite 5 enlarged and situated close to the midline. Male pygidium (tergite VIII) and part of propygidium (tergite VII) exposed; pygidium ca. 0.8× as long as wide; in dorsal view, transverse, edge completely convex, forming a distinct rim around disc, apical edge straight, not arched, with an incurved tongue-like process (Figs 26, 33), being an abrupt internal projection of apical pygidium margin; in lateral view, pygidium disc weakly convex, coarsely punctured and sparsely pubescent with short hairs.

***Legs***. Femora incrassate, with 1+3–4 teeth (usually 3). Tibiae more or less straight, slightly longer than femora; pro- and mesotibia mucronate, metatibia unarmed in male, all tibiae unarmed in female. Tarsi robust, tarsomere 1 elongate, tarsomere 2–3 transverse. Claws basally fused.

***Male genitalia and terminalia***. Aedeagal tube depressed, in lateral view pedon almost straight, apex weakly incurved; in dorsal view, pedon with apex subrounded or blunt-tipped, symmetrical, tectum narrowed and basally fused to pedon but articulated with temones. Temones normal or extremely elongate. Endophallus with small dense denticles and a rather to extremely elongate flagellum at least longer than penis, spirally coiled together with similarly elongate temones in abdomen of *Z. rhymbon*, base of flagellum slightly incrassate, a large sclerite visible. Tegminal plate flat to moderately enveloping; dorsal plate with apex deeply or not notched, apical margin constricted, bearing moderately dense long apical setae on each lobe; fenestrae variable, well marked and widely closed internally in *Z. lophos*, or open externally and contiguous internally in *Z. rhymbon*. Prostegium moderately to distinctly protruding, apex widely bifurcate or roundly constricted apicad. Manubrium extremely elongate or not, apex not broadened, undeveloped. Ninth sternite (spiculum gastrale) asymmetrical, bowknot-shaped, winged, manubrium narrow and distinctly elongate.

***Sexual dimorphism***. Female externally different from male by relatively to distinctly elongate, glabrous prorostrum and all tibiae not mucronate.

#### Distribution.

China (Yunnan); Thailand.

#### Etymology.

This genus is dedicated to Prof. Zhang Runzhi, who leads and supports a group to contribute on weevil systematic of Chinese Fauna.

##### Key to species of *Zhangius* gen. nov. (male)

**Table:** 

1	Body length 2.30–2.40 mm; rostrum with dispersed scales in rows and tip of rostrum scarcely squamose; in dorsal view, sides of prorostrum tapering apicad; each elytron with 5 suberect blackish fascicles of scales arranged in V shapes on interstriae 1, 3, 4, 5 and 7 respectively in basal area; aedeagal temones and flagellum both extremely elongate, spirally coiled, flagellum ca. 4.86× as long as aedeagal tube	***Z. rhymbon* sp. nov**.
–	Body length 2.50–2.75 mm; rostrum with relatively dense scales not in rows and prorostrum almost completely squamose; in dorsal view, sides of prorostrum parallel; each elytron with 3 suberect blackish fascicles of scales arranged in V respectively on interstriae 1, 3, 5 at base, on interstria 3 another tuft of scales located at middle area of elytra; only aedeagal flagellum distinctly elongate, ca. 2.21× as long as temones, 1.44× as long as aedeagal tube, aedeagal temones normal, short	***Z. lophos* sp. nov**.

##### Key to species of *Zhangius* gen. nov. (female)

**Table:** 

1	Prorostrum slightly elongate, shorter than metarostrum, conical, squamose	***Z. rhymbon* sp. nov**.
–	Prorostrum distinctly elongate, longer than metarostrum, tubiform, only tip slightly squamose	***Z. lophos* sp. nov**.

### Zhangius rhymbon

Taxon classificationAnimaliaColeopteraBrentidae

Wang & Alonso-Zarazaga sp. nov.

479C28FA-17E6-5E30-90C3-906BC8AE1844

https://zoobank.org/4C5A6DE2-9247-49FE-97CD-B7164E6318B1

Figs 1–3, 6, 7, 10, 11, 16–27, 35–37

#### Description

**(holotype, male). *Measurements*** (mm): standard length: 2.40. ***Rostrum***: length: 0.92, maximum width: 0.20. ***Pronotum***: median length: 0.80, maximum width: 1.30. ***Elytra***: median length: 1.98, maximum width: 1.45.

***Integument*** with dorsal part generally reddish to dark brown, ventral part slightly paler, legs yellowish to pale brown with dark brown marks on femora distally and middle tibiae, meso- and metarostrum dark brown with shiny blackish keels, prorostrum reddish brown, antennae pale brown except the 3^rd^ club segment dark brown.

***Vestiture*** composed of whitish, yellowish, brownish and black, lanceolate scales, those on antennae, rostrum and legs slightly narrower than elsewhere; rostrum with scales dense, dorsal scales semitransparent to brownish, lateral and ventral scales white, several evidently elongate scales located at lateral prorostrum, about 2× as long as other normal scales; suberect blackish scales arranged in fascicles respectively on interstriae 1, 3, 4, 5 and 7, that on interstriae 1 located at about basal 1/3 of elytra, while the other tufts gradually closer to the elytral base from interstriae 1 to 7, forming a V-shaped band, scale dark brown, apex curved backward; behind the fasciation following another white V-shaped band; other scales on interstriae not fasciate; middle area of ventral part include inner sides of coxae with soft elongate hairs, especially dense at ventrites; specialized setae present on odd interstriae and pronotum, short.

***Rostrum*** (Figs 16, 17) subcylindrical; in dorsal view: ca. 4.58× as long as maximum width, ca. 1.15× as long as pronotum in midline, widest at mesorostrum; prorostrum slightly constricted apicad, apex shiny with tiny punctures; mesorostrum with two strong dorsal submedial keels, protuberant and shiny black, dorsal submedial sulci wide, sparsely punctate; metarostrum with sides almost parallel, dorsal medial keels present, dorsal submedial sulci and dorsal submedial keels weak, wrinkled with irregular longitudinal short keels; in lateral view: nearly straight, prorostrum sides converging to apex; keels and sulci clear, scrobe enlarged and almost fused with false scrobe, parallel with rostral longitudinal axis.

***Head*** subconical, almost as long as wide; frons flat, not forming an angle with metarostrum in lateral view; eyes subrounded, flat, not touching in dorsal midline.

***Antennae*** inserted at basal 0.63 of rostral length, scape ca. 6.00× as long as wide, ca. 2.50× as long as mesorostral width, ca. 1.35× as long as club, ca. 1.17× as long as funicle; funicle with 6 desmomeres, desmomere 1 symmetrical, desmomeres 2–6 slightly asymmetrical, 1^st^ ca. 2.00× as long as wide, 2^nd^ ca. 1.67× as long as wide, 3^rd^ ca. 1.50× as long as wide, 4^th^ ca. 1.00× as long as wide, 5^th^ ca. 1.00× as long as wide, 6^th^ ca. 0.80× as long as wide; club loose, elongate, ca. 2.93× as long as wide, widest at base of 3^rd^ segment.

***Pronotum*** trapezoidal, moderately transverse, in dorsal view ca. 0.62× as long as wide, slightly widened at middle, widest at base, base ca. 2.10× as wide as apex, apical margin straight, basal margin slightly projecting posteriad at middle; in lateral view, clearly prominent at middle, nearly forming an obtuse angle; discal punctures dense and fine.

***Elytra*** elongate, triangular, evidently constricted apicad, ca. 1.37× as long as wide, ca. 2.48× as long as pronotum, in dorsal view maximum width across humeri, base teeth of elytra indistinct; dorsal surface with tubercles at basal interstriae 1, 3, 4, 5, humeri at interstria 7 also developed, tubercle at interstriae 1 elongate along elytral suture, larger than others, located at basal 1/3 of elytra; striae shallow but clear, punctate. Interstriae flat with fine punctures, interstria 8 basally with crenulate keel not surpassing humeri.

***Ventral areas***. Procoxae projecting, subconical, tangential with respect to each other. Mesocoxae separated by a distance less than 1/2 transverse diameter of mesocoxa. Abdominal ventrites densely and finely punctate, suture I invisible, suture IV complete, ventrites 3–5 midlaterally foveate, foveae of ventrite 3 reach to sides, foveae of ventrite 4 widened and conjoint medially, foveae of ventrite 5 enlarged and situated close to midline. Pygidium (tergite VIII) and part of propygidium (tergite VII) exposed, ca. 0.82× as long as wide; in dorsal view, transverse, edge completely convex, forming a distinct rim around disc, apical edge straight, not arched, with an incurved tongue-like process; in lateral view, pygidium disc weakly convex, coarsely punctured and sparsely pubescent with short hairs.

***Legs***. Femora incrassate, with 1+3–4 teeth (usually 3), profemur ca. 2.57× as long as wide, ca. 0.90× as long as protibia, widest at middle. Tibiae almost straight, protibia ca. 8.33× as long as wide; pro- and mesotibia mucronate, metatibia unarmed. Tarsi robust, protarsomere 1 ca. 1.71× as long as wide, tarsomere 2 ca. 1.11× as long as wide, tarsomere 3 bilobed, ca. 0.67× as long as wide, lobes slightly flat, ca. as wide as onychium in dorsal view, onychium ca. 5.20× as long as wide, projecting from lobes of tarsomere 3 for ca. 1.17× its length.

***Genitalia and terminalia***. Aedeagal tube depressed, in lateral view pedon almost straight, apex weakly incurved, in dorsal view pedon with sides slightly widened apicad, apex subrounded, symmetrical; tectum narrow, sides parallel, apex slightly constricted, rounded, basally fused with pedon, articulated with temones. Temones rather elongate, ca. 2.86× as long as aedeagal tube. Endophallus with small dense denticles and an extremely elongate flagellum spirally coiled together with temones in abdomen, ca. 4.86× as long as aedeagal tube, ca. 1.70 as long as temones, base of flagellum slightly incrassate, one large sclerite visible. Tegminal plate with sides moderately enveloping, ca. 0.38× as long as manubrium; Parameroid lobes apically deeply notched, apical margin constricted, bearing moderately dense long apical setae on each lobe. Fenestrae open externally and contiguous internally. Prostegium articulated with free ring, distinctly protruding with apex slightly bifurcate, divided into 2 short and blunt projections, protracted outward. Manubrium extremely elongate, apex undeveloped, ca. 9.28× as long as free ring. Ninth sternite (spiculum gastrale) asymmetrical, bowknot-shaped, winged, manubrium ca. 6.67× as long as arms.

#### Variation.

**Male paratypes**. Measurements (*N* = 4): Standard length: 2.30–2.40. Rostrum: length: 0.88–0.92, maximum width: 0.22–0.24. Pronotum: median length: 0.78–0.80, maximum width: 1.25–1.30. Elytra: median length: 1.89–1.98, maximum width: 1.40–1.45.

**Female paratypes**. Measurements (*N* = 5): Standard length: 2.25–2.50. Rostrum: length: 0.88–0.95, maximum width: 0.23–0.24. Pronotum: median length: 0.76–0.82, maximum width: 1.30–1.35. Elytra: median length: 1.95–2.10, maximum width: 1.50–1.55. Sexual dimorphism weak; females differ from males by moderately longer and constricted prorostrum, all tibiae not mucronate.

#### Materials.

***Holotype***. • ♂ (white, printed): 云南西双版纳勐仑 [Yúnnán, Xīshuăngbǎnnà, Měnglún]/ 巴卡小寨子 [Bǎkǎxiǎozhaìzǐ], 880 m/2006.VIII.6, leg. 郑国 [Zhèng Guó]// (white, printed): IOZ(E)1798272, deposited in IZCAS. This is the Bakaxiaozhaizi Village (21.964111°N, 101.210785°E) at Menglun Town, Mengla County, Jinghong City, Yunnan (P. R. of China). ***Paratypes*** (17♂ 23♀): • 1♂ 3♀, 云南西双版纳勐仑植 [Yúnnán, Xīshuăngbǎnnà, Měnglún, Zhí-/ 物园绿石林 [-wùyuán, Lǜshílín], 643 m/2007.VII.18, leg. 唐果 [Táng Guŏ]// (white, printed): IOZ(E)1639773, IOZ(E)1798276, IOZ(E)1639706, IOZ(E)1639599, deposited in IZCAS. • 3♂ 2♀, (white, printed): 云南西双版纳勐仑 [Yúnnán, Xīshuăngbǎnnà, Měnglún]/ 巴卡小寨子 [Bǎkǎxiǎozhaìzǐ], 880 m/2006.VIII.6, leg. 郑国 [Zhèng Guó]// (white, printed): IOZ(E)1798274, IOZ(E)1798275, IOZ(E)1798277, IOZ(E)1798278, deposited in IZCAS. • 2♂, 云南西双版纳勐仑植物园55 [Yúnnán, Xīshuăngbǎnnà, Měnglún, Zhíwùyuán 55-]/ 公里样地 [-gōnglǐyàngdì], 2014.IV.5, leg. 王志良 [Wáng Zhìliáng], deposited in IZCAS. • 3♂ 3♀: (white, printed): N-THAILAND, 2004/72 km N Chiana Mai/ Chiana Dao env. (should be Chiang Dao County of Chiang Mai)/F. Pavel leg., 26–29.4., deposited in coll. M. Wanat, MNHW. • 5♂ 12♀, 云南西双版纳植物园 [Yúnnán, Xīshuāngbǎnnà, Zhíwùyuán], leg. 王志良 [Wáng Zhìliáng], 2016.VII.25, deposited in MBFU. • 1♂ 1♀, 云南西双版纳植物园 [Yúnnán, Xīshuāngbǎnnà, Zhíwùyuán], leg. 王志良 [Wáng Zhìliáng], 2016.VII.25, deposited in MNHN. • 1♂ 1♀, 云南西双版纳植物园 [Yúnnán, Xīshuāngbǎnnà, Zhíwùyuán], leg. 王志良 [Wáng Zhìliáng], 2016.VII.25, deposited in SMTD. • 1♂, (white, printed): 云南西双版纳勐仑 [Yúnnán, Xīshuăngbǎnnà, Měnglún]/巴卡小寨子 [Bǎkǎxiǎozhaìzǐ], 880 m/2006.VIII.6, leg. 郑国 [Zhèng Guó]// (white, printed): • ♂//(orange, printed and handwritten): Paratypus/ Zhangius/ rhymbon/ Wang &/ Alonso-Z. des. 2025// (gray-blue, printed): MNCN_Ent/255916, deposited in coll. Alonso-Zarazaga, MNCN. • 1 ♀, 云南西双版纳勐仑植 [Yúnnán, Xīshuăngbǎnnà, Měnglún, Zhí-/ 物园绿石林 [-wùyuán, Lǜshílín], 643 m/2009.XI.17, leg. 唐果 [Táng Guŏ]// (white, printed): • ♀//(orange, printed and handwritten): Paratypus/ Zhangius/ rhymbon/ Wang &/ Alonso-Z. des. 2025//(gray-blue, printed): MNCN_Ent/255917, deposited in coll. Alonso-Zarazaga, MNCN.

#### Distribution.

China (Yunnan), Thailand.

#### Etymology.

This species is named “rhymbon” (coil of a serpent) after the shape of its extremely elongate flagellum, aedeagus and tegmen, which are together curled in abdomen. It is a Latinized Greek noun in apposition.

### Zhangius lophos

Taxon classificationAnimaliaColeopteraBrentidae

Wang & Alonso-Zarazaga sp. nov.

4B82D4B7-FDF6-5705-9F9C-9DFB6A63D7F2

https://zoobank.org/FEF3F1F8-CDB6-44C1-8D6D-18AAB5786787

Figs 8, 9, 12–15, 28–34

#### Description

**(holotype, male). *Measurements*** (mm): Standard length: 2.50. ***Rostrum***: length: 1.10, maximum width: 0.22. ***Pronotum***: median length: 0.92, maximum width: 1.40.

***Integument*** with dorsal part generally reddish to dark brown, ventral part reddish brown, legs yellowish except distal 1/2 of femora and basal 1/2 of tibiae dark brown, meso- and metarostrum dark brown with blackish keels, prorostrum and antennae pale to reddish brown.

***Vestiture*** composed of whitish, yellowish to brownish and black, lanceolate scales, those on antennae narrower; rostrum with dense, a little elongate, yellow to brownish scales; pronotum with clearly dense, pure brownish and centripetal vestiture; interstriae 1, 3, 5 and 7 alternately with blackish and whitish or brownish scales from elytral base to declivity, scales on interstriae 2 and 4 whitish to brownish, on interstriae 6 and 8 almost blackish, on interstriae 9–10 brownish; scales on declivity whitish to brownish, each elytra with 3 suberect blackish fascicled scales arranged in V-shaped respectively on interstriae 1, 3, 5 at base, at same interstriae 3 more tufts of scales located at middle area of elytra, fascicled scales on interstriae 1 evidently elongate, located at about basal 1/3 of elytra; middle area of ventral part including inner sides of coxae with soft elongate hairs, especially dense at ventrites; specialized setae present on odd interstriae and pronotum, short.

***Rostrum*** (Figs 12, 13) subcylindrical (prorostrum) to subrectangular in section (metarostrum); in dorsal view, ca. 5.00× as long as maximum width, ca. 1.20× as long as pronotum in midline, widest at mesorostrum; prorostrum not constricted apicad, apex shiny with tiny punctures; mesorostrum with two strong dorsal submedial keels, protuberant and shiny, dorsal submedial sulci wide, surface longitudinally wrinkled; metarostrum with sides almost parallel, dorsal medial keel developed, dorsal submedial sulci and dorsal submedial keels weak, longitudinally wrinkled with irregular short keels; in lateral view, almost straight, keels and sulci clear, scrobe enlarged and almost fused with false scrobe, parallel with rostral longitudinal axis.

***Head*** subconical, almost as long as wide; frons flat, not forming an angle with metarostrum in lateral view; eyes subrounded, enlarged, flat, not touching in dorsal midline.

***Antennae*** inserted at basal 0.55 of rostral length, scape ca. 7.71× as long as wide, ca. 2.45× as long as mesorostral width, ca. 1.35× as long as club, ca. 1.04× as long as funicle; funicle with 6 desmomeres, desmomere 1 symmetrical, desmomeres 2–6 slightly asymmetrical, 1^st^ ca. 2.00× as long as wide, 2^nd^ 2 ca. 2.22× as long as wide, 3^rd^ ca. 1.60× as long as wide, 4^th^ ca. 1.60× as long as wide, 5^th^ ca. 0.75× as long as wide, 6^th^ ca. 1.00× as long as wide; club loose, elongate, ca. 2.86× as long as wide, widest at base of the 3^rd^ segment.

***Pronotum*** trapezoid, moderately transverse; in dorsal view, ca. 0.66× as long as wide, slightly widened at middle, evidently widest at base, base ca. 2.41× as wide as apex, apical margin straight, basal margin slightly projecting posteriad at middle; in lateral view, distinctly prominent at middle, almost forming a right angle; discal punctures dense and fine.

***Elytra*** elongate, triangular, evidently constricted apicad, ca. 1.45× as long as wide, ca. 2.39× as long as pronotum; in dorsal view, maximum width across humeri, base teeth of elytra indistinct; disc with tubercles at basal interstriae 1, 3, 4, 5, humeri also well developed, tubercle at interstriae 1 elongate along elytral suture, larger than others, located at basal 1/3 of elytra; striae shallow but clear, punctate; interstriae flat, finely punctured, interstriae 8 basally crenulate-keeled, not surpassing humeri.

***Ventral areas***. Procoxae projecting, subconical, tangent. Mesocoxae separated by a distance less than 1/2 transverse diameter of mesocoxa. Abdominal ventrites densely and finely punctured, suture I invisible, suture IV complete, ventrites 3–5 midlaterally foveate, foveae of ventrite 3 not reaching to sides and transversely closed (medial margin distinct), foveae of ventrite 4 widened and medially opened (medial margin distinct), foveae of ventrite 5 enlarged and situated close to the middle line of anterior margin, another two shallow and narrow foveae along sides. Pygidium (tergite VIII) and part of propygidium (tergite VII) exposed, ca. 0.81× as long as wide, in dorsal view, transverse, edge slightly convex, rim weak, apical edge straight, not arched, with an incurved tongue-like process; in lateral view, pygidium disc weakly convex, coarsely punctured and sparsely pubescent with short hairs.

***Legs***. Femora incrassate, with 1+3–4 teeth (irregular), ca. 2.50× as long as wide, ca. 0.85× as long as protibia, widest at middle. Tibia almost straight at outer surface, inner surface slightly sinuated; protibia ca. 7.87× as long as wide; pro- and mesotibia mucronate, metatibia unarmed. Tarsi robust, protarsomere 1 ca. 2.15× as long as wide, 2 ca. 1.32× as long as wide, 3 bilobed, ca. 0.74× as long as wide, lobes slightly flat, as wide as onychium in dorsal view, onychium ca. 5.45× as long as wide, projecting from lobes of tarsomere 3 for ca. 1.14× its length.

***Genitalia***. Aedeagal tube depressed, in lateral view pedon almost straight, apex weakly incurved, in dorsal view, pedon with sides slightly constricted medially, apex blunt, symmetrical; tectum narrow, sides parallel, medially sclerotized, apex slightly constricted, subrounded, basally fused with pedon, articulated with temones. Temones short, ca. 0.65× as long as aedeagal tube. Endophallus with small dense denticles and a distinctly elongate flagellum, ca. 1.44× as long as aedeagal tube, ca. 2.21× as long as temones, base of flagellum clearly incrassate, 2 large frena visible. Tegminal plate almost flat, sides weakly curved ventrally, ca. 2.36× as long as manubrium. Parameroid lobes apically unnotched, apical margin constricted and protruding, bearing moderately dense long apical setae along the margin. Fenestrae open externally and widely closed internally. Prostegium evidently protruding medially, apex nearly truncate, articulated with free ring. Manubrium short with apex slightly broadened, ca. 0.93× as long as free ring. Ninth sternite (spiculum gastrale) asymmetrical, bowknot-shaped, winged, manubrium ca. 4.25× as long as arms.

#### Variation.

**Male**. Measurements (*N* = 4): standard length: 2.50–2.75: Rostrum: length: 1.10–1.30, maximum width: 0.22–0.24. Pronotum: median length: 0.85–0.92, maximum width: 1.40–1.55. Elytra: median length: 2.20–2.35, maximum width: 1.52–1.60. **Female**. Standard length: 2.50–2.70. Rostrum: length: 1.30–1.56, maximum width: 0.24–0.25. Pronotum: median length: 0.88–0.91, maximum width: 1.45–1.65. Elytra: median length: 2.10–2.45, maximum width: 1.62–1.68. Sexual dimorphism clear, females differ from males by the distinctly elongate, tubiform prorostrum, and all tibiae not mucronate.

#### Distribution.

China (Yunnan).

#### Materials.

***Holotype***. • ♂: (white, printed): 西双版纳勐仑 [Yúnnán, Xīshuăngbǎnnà, Měnglún, Zhí-]/ 物园绿石林 [-wùyuán, Lǜshílín], 643 m, 2009.XI.20, leg. 唐果 [Táng Guǒ], 姚志远 [Yáo Zhìyuán], deposited in IZCAS. Lvshilin (21.917861°N, 101.269343°E) is a scenic area located in the southeast of Xishuangbanna Tropical Botanical Garden at Menglun Town, Mengla County, Jinghong City, Yunnan (P. R. of China). ***Paratypes*** (5♂ 4♀): • 4♂ 3♀ (white, printed): 西双版纳勐仑植 [Yúnnán, Xīshuăngbǎnnà, Měnglún, Zhí-]/物园绿石林 [-wùyuán, Lǜshílín], 643 m, 2009.XI.17, leg. 唐果 [Táng Guǒ]// (white, printed): IOZ(E)1798280, IOZ(E)1798281, IOZ(E)1798282, IOZ(E)1798283; IOZ(E)1639790, IOZ(E)1639677; deposited in MBFU. • 1♂ 1 ♀ (white, printed): 西双版纳勐仑植 [Yúnnán, Xīshuăngbǎnnà, Měnglún, Zhí-]/物园绿石林 [-wùyuán, Lǜshílín], 643 m, 2009.XI.17, leg. 唐果 [Táng Guǒ]// (white, printed): • ♂ or ♀//(orange, printed and handwritten): Paratypus/ Zhangius/ lophos/ Wang &/ Alonso-Z. des. 2025//(gray-blue, printed): MNCN_Ent/255918, MNCN_Ent/255919; deposited in coll. Alonso-Zarazaga, MNCN.

#### Etymology.

This species is named “lophos” (crest of a helmet) after the clearly visible crest of hairs on the elytra in side view. It is a Latinized Greek noun in apposition.

## Discussion

The new genus *Zhangius* is to be considered one of the most ancestral in the subfamily Nanophyinae, due to the six desmomeres, the complete tenth elytral stria and the long flagellum. It is placed as a sister taxon of *Shiva* Pajni & Bhateja, 1982 (a polyphyletic taxon) by Xuankun Li et al. (unpubl.). In the key to the genera with six desmomeres (Alonso-Zarazaga and Perrin 2011) it runs to *Lyalia* Alonso-Zarazaga & Perrin, 2011, from which it differs by the setae arranged in fascicles, the elytral tubercles, the 8^th^ elytral interstria with a crenulate keel up to the end of the humeral callus, the male metatibiae not mucronate, and the abdominal suture I absent.

This new genus *Zhangius* seems to be linked to the plant genus *Lagerstroemia* (Lythraceae), as are several other genera of Nanophyinae. Primitive and advanced genera of this subfamily are linked to Lythraceae (*Lagerstroemia* and other genera) and other Myrtales, which can be considered a plesiomorphic trait.

## Supplementary Material

XML Treatment for Zhangius

XML Treatment for Zhangius rhymbon

XML Treatment for Zhangius lophos

## Additional information

### Use of AI

No use of AI was reported.

### Author ORCIDs

Zhiliang Wang https://orcid.org/0000-0001-6006-5402

Miguel A. Alonso-Zarazaga https://orcid.org/0000-0002-6991-0980

### Data availability

All of the data that support the findings of this study are available in the main text.
